# Enhancing Surgical Task Adherence Through a Rewards-Driven Mobile Application: A Single-Arm Intervention Feasibility Study

**DOI:** 10.7759/cureus.60950

**Published:** 2024-05-23

**Authors:** Pemla Jagtiani, Paul G Mastrokostas, Sean Inzerillo, Simone A Betchen

**Affiliations:** 1 School of Medicine, State University of New York Downstate Health Sciences University, New York, USA; 2 Neurological Surgery, Maimonides Medical Center, New York, USA

**Keywords:** omnichannel communication, postoperative instructions, preoperative adherence, monetary incentives, neurosurgery

## Abstract

Introduction: Ensuring patients follow preoperative and postoperative instructions is vital for maximizing surgical success. This pilot study investigates the feasibility of using monetary incentives through a nudge engine application-based model of omnichannel communication to prompt adherence to preoperative and postoperative instructions.

Methods:Over a six-month period, we conducted a longitudinal study employing the TheraPay^®^ Rewards app at Maimonides Medical Center in Brooklyn, United States. Our recruitment efforts targeted English and Spanish-speaking patients with smartphones through in-person visits and phone calls. Participants received a $15 credit on a gift card for each completed task. The tasks included preoperative validations such as obtaining primary care physician clearance, completing preoperative assessments, undergoing preoperative scans with accompanying compact disks (CDs), and discontinuing specific medications. Postoperative validations included attending postoperative visits, proper incision care, discontinuation of narcotics at three weeks, and initiation of the first physical therapy session.

Results:We enrolled 16 patients with a mean age of 59.5 years (SD 11.68), the majority being male (n = 10, 62.5%). Preoperatively, task completion rates ranged from 83% to 100%. Postoperatively, rates varied from 20% to 100%. Preoperative task adherence averaged at 98.7% (SD 2.2%), while postoperative adherence averaged 60% (SD 21%).

Conclusion: Our study indicates that financial incentives delivered through a gamified approach effectively encourage patients to complete essential preoperative tasks, suggesting a promise for enhancing adherence. Nonetheless, the decrease in postoperative task adherence highlights the necessity for careful implementation. Future investigations should compare cancellation rates and outcome measures to gain deeper insights into the effectiveness of app-based incentives in improving surgical outcomes and patient adherence.

## Introduction

Achieving a safe and efficient surgical experience requires the optimization of a patient's condition [1]. This involves securing clearance from a primary care provider (PCP), completing thorough preoperative assessments (PAT), undergoing relevant preoperative scans along with accompanying CDs, and discontinuing specific medications, collectively minimizing adverse perioperative outcomes [1-3]. The importance of adhering to postoperative monitoring is also linked to more positive outcomes [4]. This includes adhering to scheduled postoperative visits, ensuring proper incision care, discontinuing narcotics within the recommended three weeks, and initiating the first physical therapy session [4]. By embracing these practices, patients can significantly contribute to their overall well-being and recovery post-surgery.

Despite these benefits, compliance remains a significant challenge across medicine, impacting surgical outcomes and healthcare efficiency. Non-compliance, particularly in completing preoperative tasks, can lead to increased cancellations and inefficient use of operating room (OR) time causing extra administrative work and significant financial losses [5]. Communicating with patients prior to surgery is vital for preparation. Surgical rescheduling and cancellation can often arise due to patient-related factors such as forgetfulness, non-adherence to medical direction, confusion in scheduling preoperative tests, logistical scheduling errors, and information breakdown. Similar issues can also be encountered in the postoperative period.

Financial reinforcement has been proven to improve medication adherence through the elimination of present bias, in which future benefit is strongly underestimated compared to a present challenge. Financial reinforcement has demonstrated its ability in longitudinal clinical trials to increase adherence, particularly in psychiatry [5,6]. We are now investigating its use to enhance compliance in an operative setting where present bias may prevent patients from completing essential perioperative tasks. The recently developed TheraPay® Rewards mobile application, designed to address the social determinants of health to improve critical care adherence, offers an opportunity to explore its effectiveness within the surgical domain. Our study aims to provide a proof of concept that TheraPay® Rewards (Reciprocity Health, Inc., Wilmington, United States) can be utilized to assess pre and postoperative protocol adherence by offering compensation and timely reminders for each task.

## Materials and methods

Ethical approval

This research was conducted within the Department of Neurosurgery at Maimonides Medical Center, New York, United States. All participants provided written informed consent as approved by the Institutional Review Board at Maimonides Medical Center (#2022-07-04).

Study design

A six-month, longitudinal study was conducted at Maimonides Medical Center to investigate the use of incentives for completing preoperative and postoperative activities among neurosurgery patients. Recruitment strategies included in-person visits and phone calls. English and Spanish-speaking patients aged 18 to 99 with a planned neurosurgery procedure and who have access to a smart device were eligible to participate in the study. The study excluded the pediatric population under 18 years of age and patients receiving non-neurosurgical care.

Upon obtaining consent, patients were guided to download the TheraPay® Rewards app. The app required patients to input their personal information, including name, email, phone number, and address. Subsequently, a rewards card, similar to a debit card, was mailed to the provided address. TheraPay® Rewards provided information on the activities patients needed to complete to earn rewards and sent daily reminders. Notably, these activities aligned with tasks that patients were required to complete regardless, both before and after surgery.

The tasks included the following preoperative validations such as obtaining PCP clearance, completing PAT, undergoing preoperative scans with accompanying CDs, and discontinuing specific medications. Postoperative validations encompassed attending postoperative visits, proper incision care, discontinuation of narcotics at three weeks, and initiation of the first physical therapy session. Patients received a monetary reward, a $15 credit per task completed, loaded onto their gift card. It's important to note that the surgical procedure itself was not considered a task for which rewards were granted. Patients were informed that the rewards card had limitations, excluding its use for certain purchases like cigarettes, alcohol, and firearms. The maximum amount loaded onto the card was $150, reflecting the cumulative rewards for successfully completing the specified pre and postoperative tasks.

Data analysis

Demographic information gathered for study participants included age and gender. Adherence was measured by the completion of tasks and reported as percentages. An intention-to-treat (ITT) analysis was utilized to investigate the use of incentives for task completion rates. The average adherence rates of the preoperative and postoperative phases were calculated using the individual task adherence rates from each respective phase. The Wilcoxon signed-rank test was employed to compare the average adherence rates between the preoperative and postoperative phases. All statistical analyses were performed using R statistical software (version 4.3.1; R Project for Statistical Computing, Vienna, Austria).

## Results

**Figure 1:**
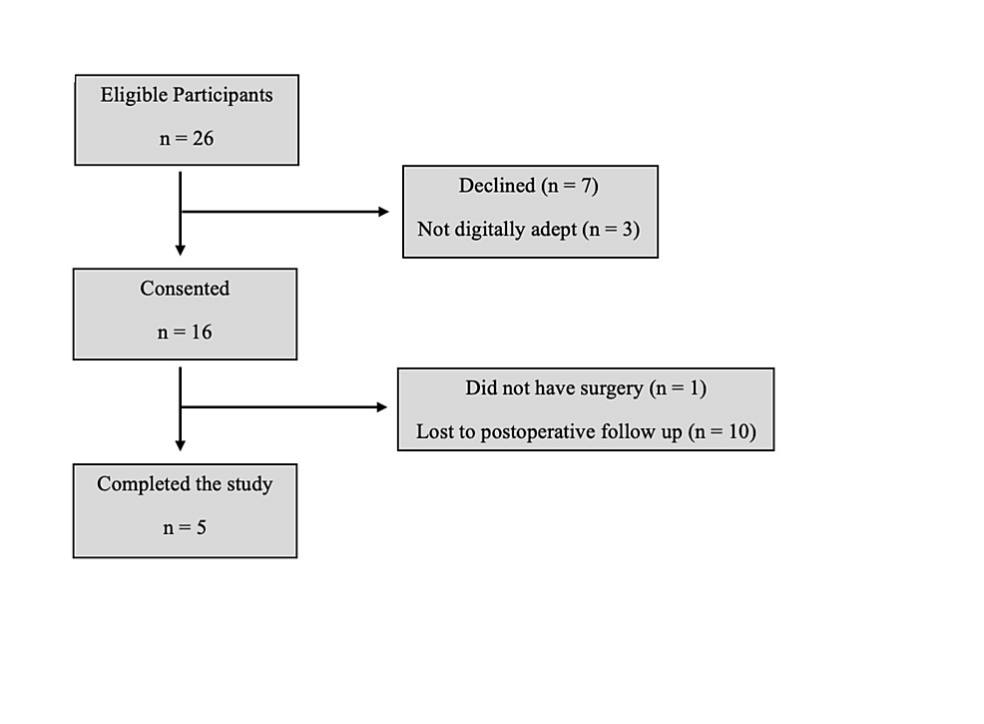
Flow chart of patient inclusion and exclusion criteria

A total of 16 patients were enrolled over a six-month period. The mean age was 59.5 years old (SD 11.68) at the time of scheduled surgery and the majority were male (n = 10, 62.5%). One patient did not complete surgery and, of the remaining sample, 10 patients (66.7%) were lost to follow-up during the two-month postoperative phase (Figure 1).

The analysis of task completion revealed high adherence rates among patients in both preoperative and postoperative phases. In the preoperative phase, completion rates ranged from 83% (5/6) to 100% (15/15) across different tasks, indicating robust adherence to preoperative protocols. Individual task adherence percentages from the preoperative phase are included in Table 1.

**Table 1:** Individual task adherence rate for the preoperative phase PCP: primary care provider; PAT: preoperative assessment; CD: compact disk

Preoperative tasks	Average adherence rate
PCP clearance (n = 16) (%, n)	88% (14/16)
PAT completion (n = 15) (%, n)	100% (15/15)
Preoperative scans and CDs (n = 15) (%, n)	100% (15/15)
Medication discontinuation (n = 6) (%, n)	83% (5/6)
Average preoperative adherence rate (%, ± SD)	98.7% ± 2.2%

Similarly, in the postoperative phase, completion rates ranged from 20% (3/15) to 93% (14/15), highlighting a varying commitment of the patients to postoperative task completion. Individual task adherence percentages from the postoperative phases are included in Table 2.

**Table 2:** Individual task adherence rate for the postoperative phase PT: physical therapy

Postoperative tasks	Average adherence rate
Postoperative visit completion (n = 15) (%, n)	93% (14/15)
Incision care completion (n = 15) (%, n)	93% (14/15)
Postoperative narcotic discontinuation (n = 15) (%, n)	33% (5/15)
Initial PT session completion (n = 15) (%, n)	20% (3/15)
Average postoperative adherence rate (%, ± SD)	60% ± 21%

The average task adherence rate for the preoperative phase was 98.7% (SD 2.2%) and the postoperative average overall adherence rate was 60% (SD 21%). A comparison of average preoperative and postoperative task adherence rates demonstrated a higher average adherence during the preoperative phase (n = 15, V = 91, p < 0.001). One patient didn’t have surgery and was removed from this paired analysis.

## Discussion

This study explored the implementation of financial incentives, facilitated through the TheraPay® Rewards mobile application, to investigate adherence to preoperative and postoperative tasks among patients undergoing neurosurgery. The initiative was part of a pilot project aiming to utilize omnichannel care coordination alongside a digital nudge engine and overlaid rewards to motivate patient adherence and ultimately drive improved health outcomes. The concept of utilizing behavioral economics, specifically financial incentives, using a mobile app to influence health-related behaviors is not novel [7,8]. However, extending this approach to the surgical domain, particularly in the context of preoperative and postoperative task adherence, offers a unique contribution to the existing literature.

The results demonstrate a high level of adherence to preoperative tasks, suggesting that app-delivered financial incentives can effectively motivate patients to complete crucial preparations for surgery. This finding aligns with previous research indicating that financial incentives can improve medication adherence [8]. The similarity in the effectiveness of financial incentives across different health behaviors emphasizes the versatility and potential of this approach to enhance patient engagement and adherence to recommended medical protocols.

The observed decrease in postoperative task adherence presents a complex challenge in app-delivered financial incentive implementation. It highlights that the motivation provided by financial incentives may not be as effective or enduring in the postoperative phase, potentially due to postoperative pain, decreased mobility, psychological factors, or a perceived reduction in the need to adhere to medical protocols following surgery. These findings suggest that while TheraPay can be a useful tool for investigating adherence, its effectiveness may vary depending on the timing and nature of the tasks [8].

To address this hurdle, integrating personalized interventions may become more common. Customizing financial incentives based on individual recovery trajectories and incorporating personalized support mechanisms, such as adaptive reminders and tailored educational or motivational messages, could bolster adherence during the more challenging postoperative period. Moreover, the modular and instantaneous nature of medical adherence apps like TheraPay® offers a robust foundation to support the implementation of individualized adherence strategies [7]. Additionally, exploring the psychological and social factors that influence patients’ responses to app-based financial incentives could offer deeper insights into how to design more effective interventions.

This study faced limitations, including a small sample size and participant dropouts, affecting the findings' generalizability. Additionally, self-reported data may have introduced social desirability bias. Recruitment was limited to English and Spanish speakers, excluding the significant proportion of Russian and Mandarin speakers in the Maimonides Medical Center patient population, reducing participant diversity. Technological challenges, particularly for older patients less familiar with mobile apps, further constrained the sample size. Future studies should expand sample sizes, include diverse language groups, and use objective completion metrics.

## Conclusions

This study investigates how financial incentives via a mobile app affect preoperative and postoperative task adherence in neurosurgery patients. Results show high preoperative compliance but reduced postoperative adherence, highlighting the potential of app-delivered incentives and the impact of task timing and characteristics on effectiveness. Future research should extend to diverse patient groups and settings to refine the use of app-based incentives in enhancing patient outcomes.
